# Supplementary figures and images for: Hexokinase II dissociation alone cannot account for changes in heart mitochondrial function, morphology and sensitivity to permeability transition pore opening following ischemia
Source: PLoS One. 2020 Jun 24;15(6):e0234653. doi: 10.1371/journal.pone.0234653 (PMC7313731; doi:10.1371/journal.pone.0234653)

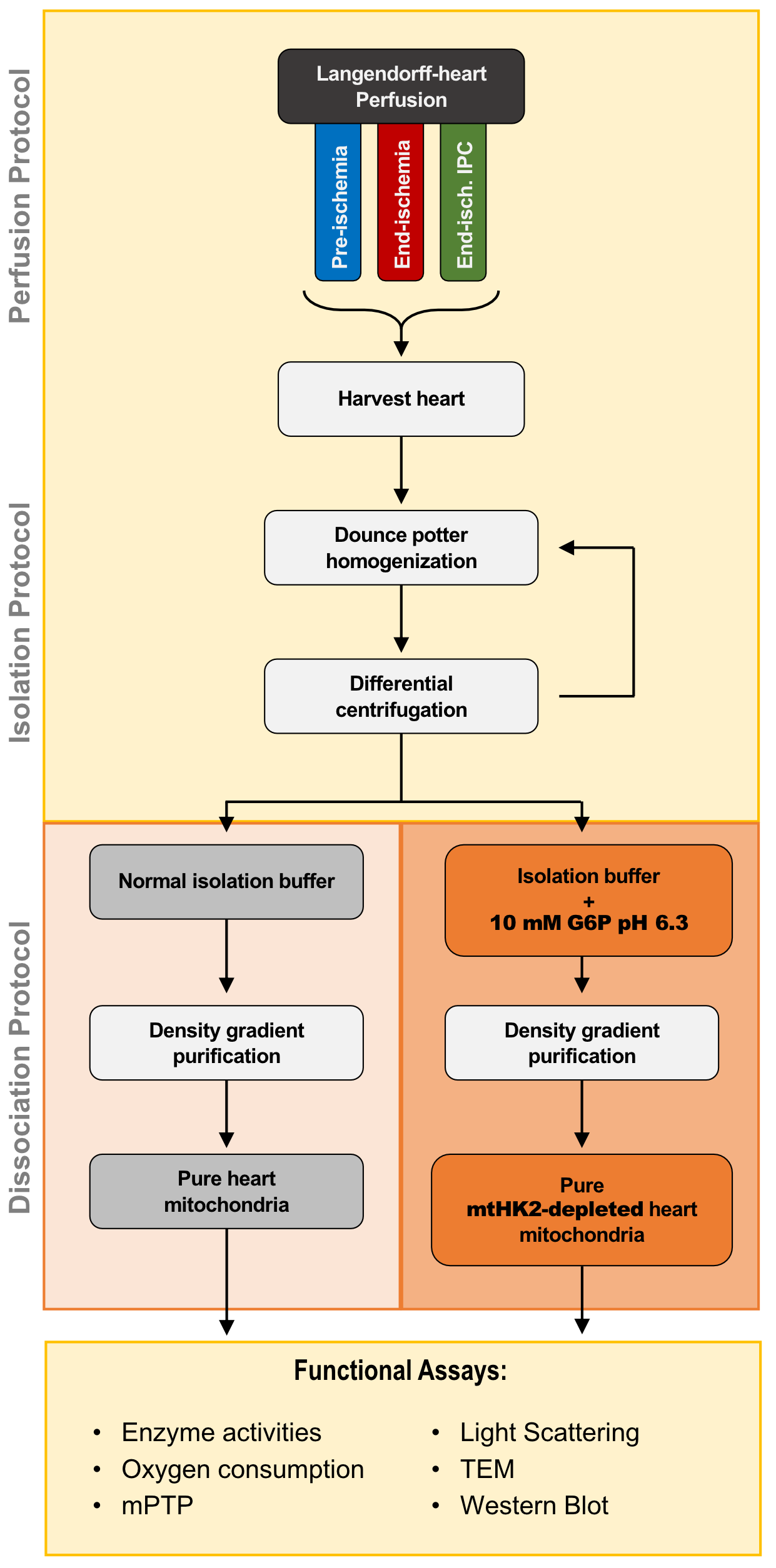

Supplement: S1 Fig — Note that the dissociation protocol used to release mitochondrial-bound HK2 occurs prior to data acquisition and that dissociation agents are absent from the washing buffers. (TIFF) [file pone.0234653.s001.tiff]

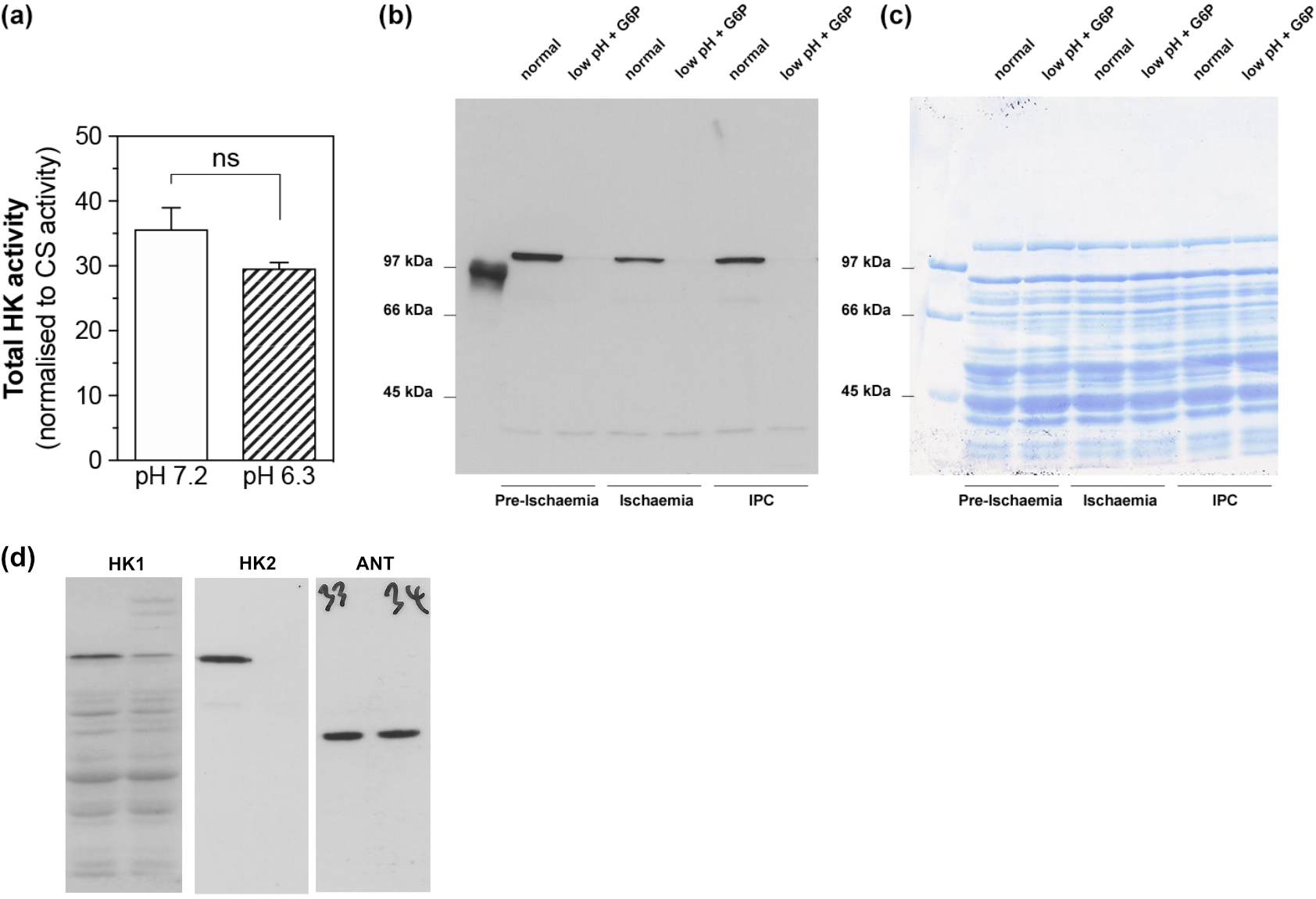

Supplement: S2 Fig — Total HK activity of mitochondria pre-treated with dissociation buffer during isolation protocol (a); data are presented as mean ± SEM of 4 independent experiments on different mitochondrial preparations. Differences between groups were evaluated by a matched pair (± low pH) t Student’s test. (b) uncropped and representative immunoblot against HK2 on isolated mitochondrial fractions. (c) Coomassie staining of membrane shown in (b). The loading control for membrane in (b) is shown on S6b Fig (the mitochondrial protein ANT). (d) uncropped immunoblots against HK2, HK1 and ANT as shown on main Fig 1. (TIFF) [file pone.0234653.s002.tiff]

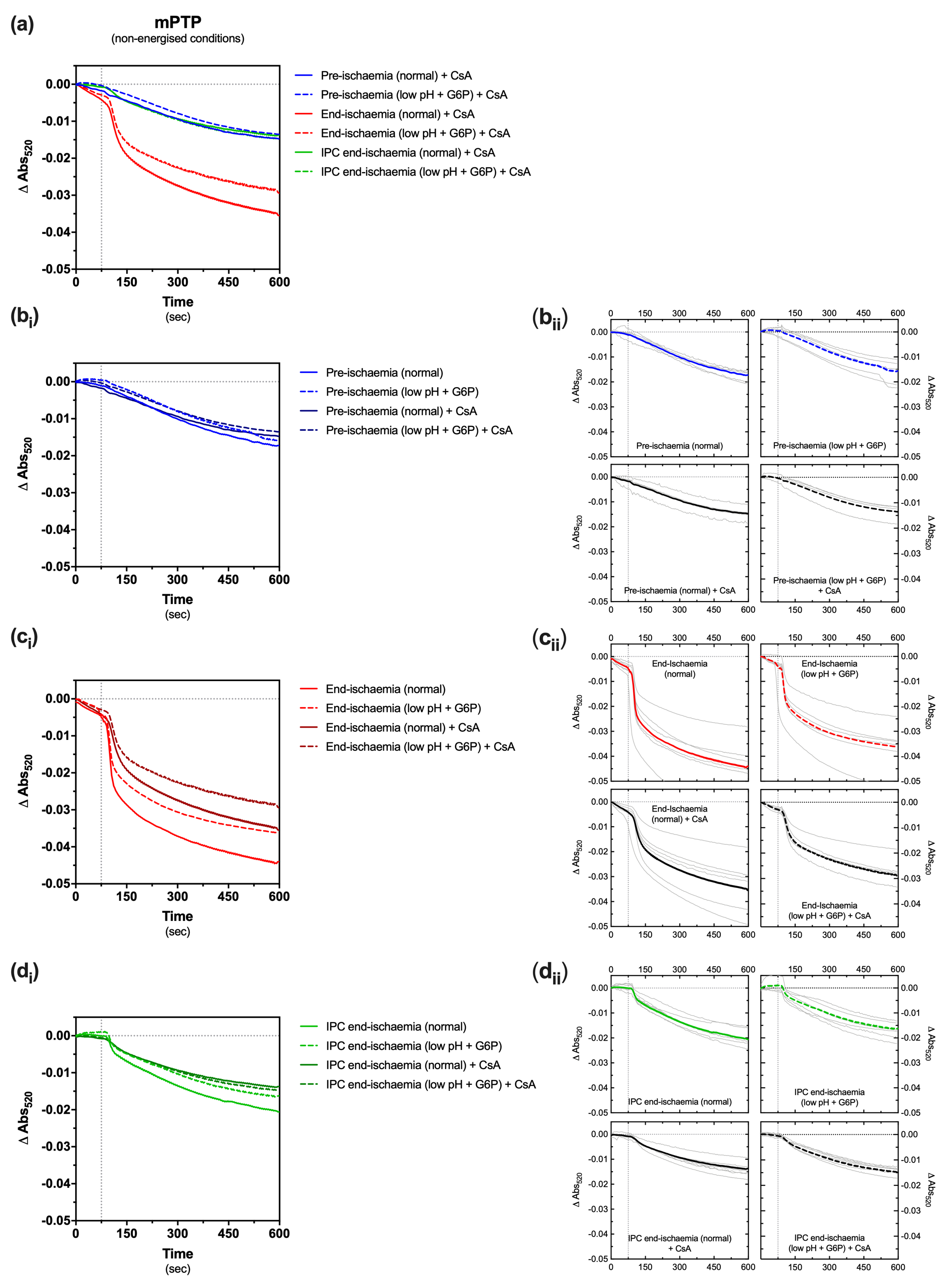

Supplement: S3 Fig — Mitochondrial swelling associated with mPTP opening was triggered by addition of 83.5 μM free Ca2+ and monitored by measurement of A520 (a). Data are presented either as mean of 5–6 independent experiments (bi-ci) or overlaid with individual runs (bii-cii) for the perfusion groups in the study (pre-ischaemia—bi, bii; end-ischaemia—ci, cii; and, IPC end-ischaemia—di, dii). (TIFF) [file pone.0234653.s003.tiff]

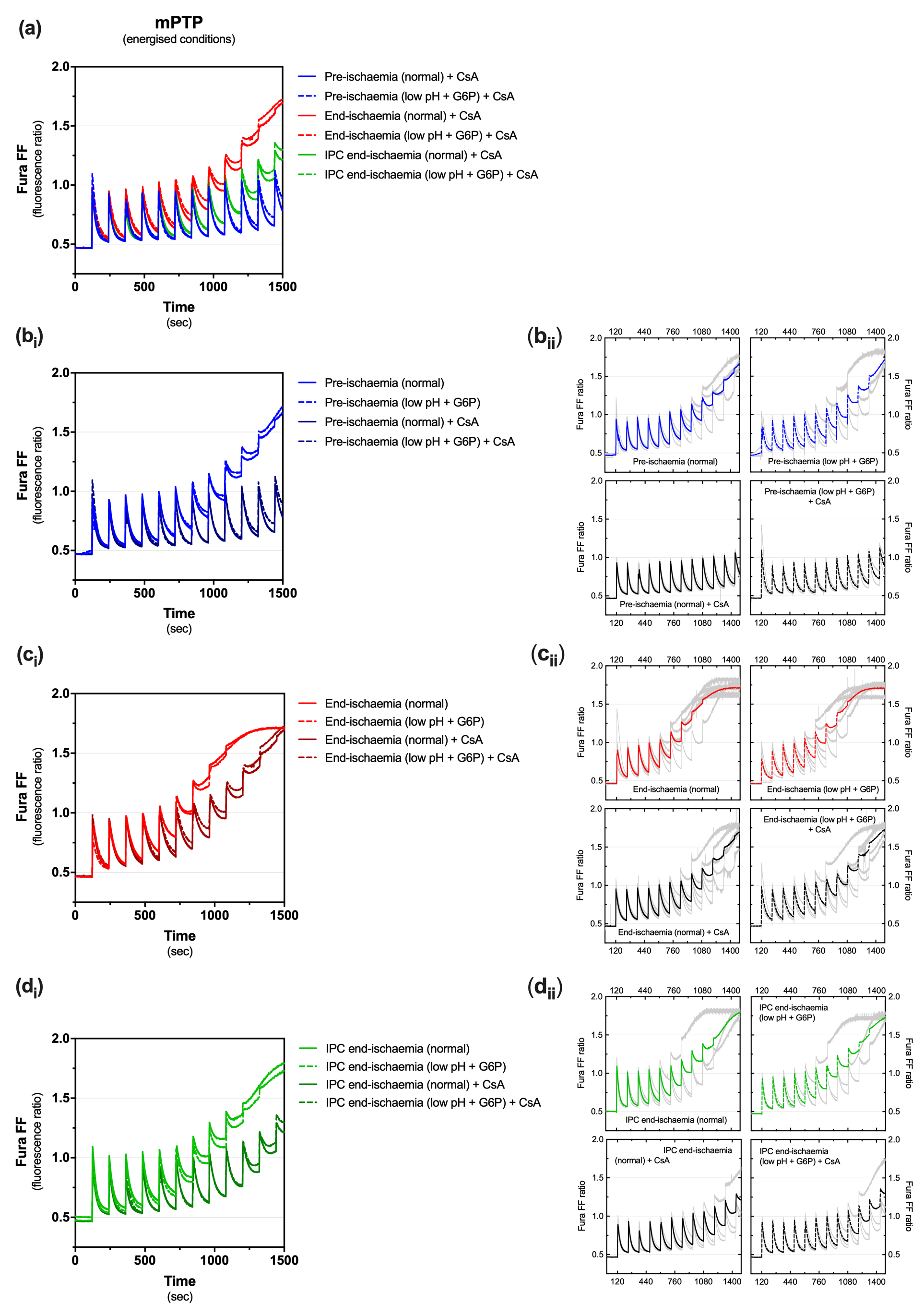

Supplement: S4 Fig — mPTP opening was evaluated by the calcium retention capacity assay with addition of 20 μM free Ca2+ every 2 min (a). Data are presented as mean independent experiments (bi-ci) or overlaid with individual runs (bii-cii) for the perfusion groups in the study (pre-ischaemia—bi, bii; end-ischaemia—ci, cii; and, IPC end-ischaemia—di, dii). (TIFF) [file pone.0234653.s004.tiff]

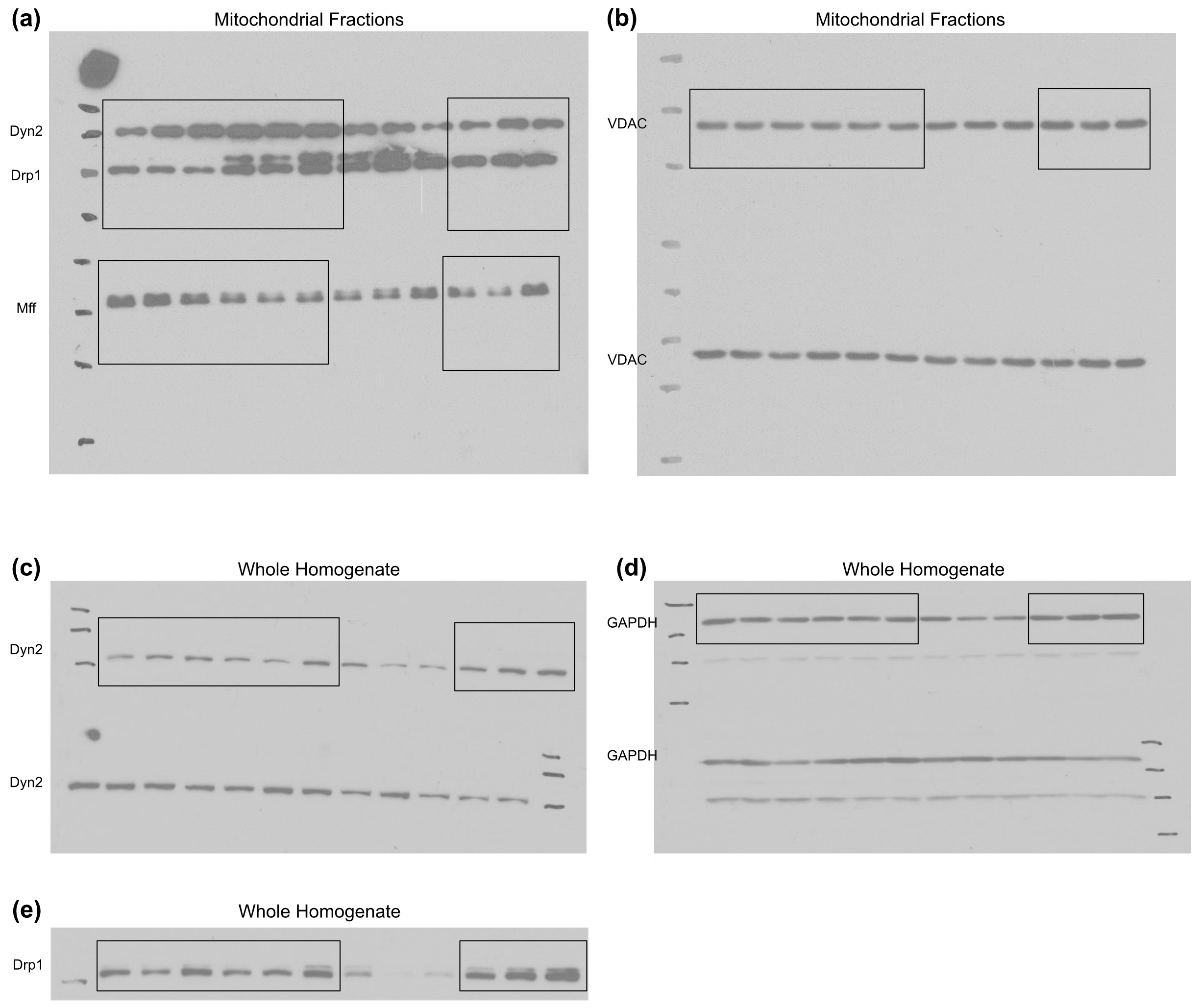

Supplement: S5 Fig — Dyn-2 (a, b), Drp1 (a, b) and Mff (c) data shown on Fig 5. Total extracts are shown in (a) while mitochondrial fractions are shown in (b), for Dyn-2 and Drp1. For Mff only mitochondrial fractions were analyzed. GAPDH was used as loading control for the total extracts while VDAC was used for mitochondrial fractions instead. The vertical white bar along the blots on (a) and (b) indicate that although the samples were in the same membrane they were not next to each other (non-relevant samples are omitted in the picture). (TIFF) [file pone.0234653.s005.tiff]

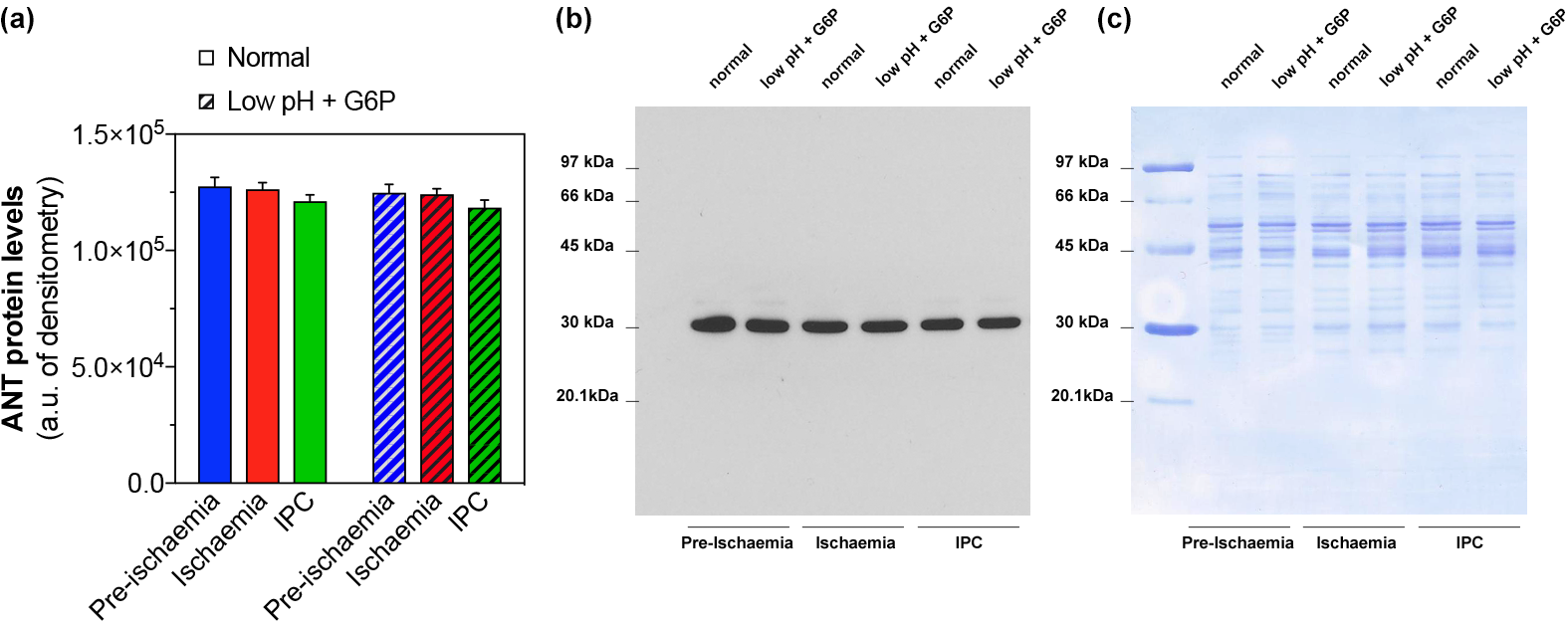

Supplement: S6 Fig — Data are presented as mean ± SEM of 6–8 independent mitochondrial fraction samples (a). For details on the use of “low pH + G6P” please see the Material and methods section. Differences between groups were evaluated by a matched pair (± G6P low pH) two-way ANOVA with interaction followed by Holm-Šídák pos-hoc test to correct for multiple comparisons. (b) representative western blot of the analysis in (a). (c) loading control of the western blot shown on (b) by Coomassie staining. (TIFF) [file pone.0234653.s006.tiff]

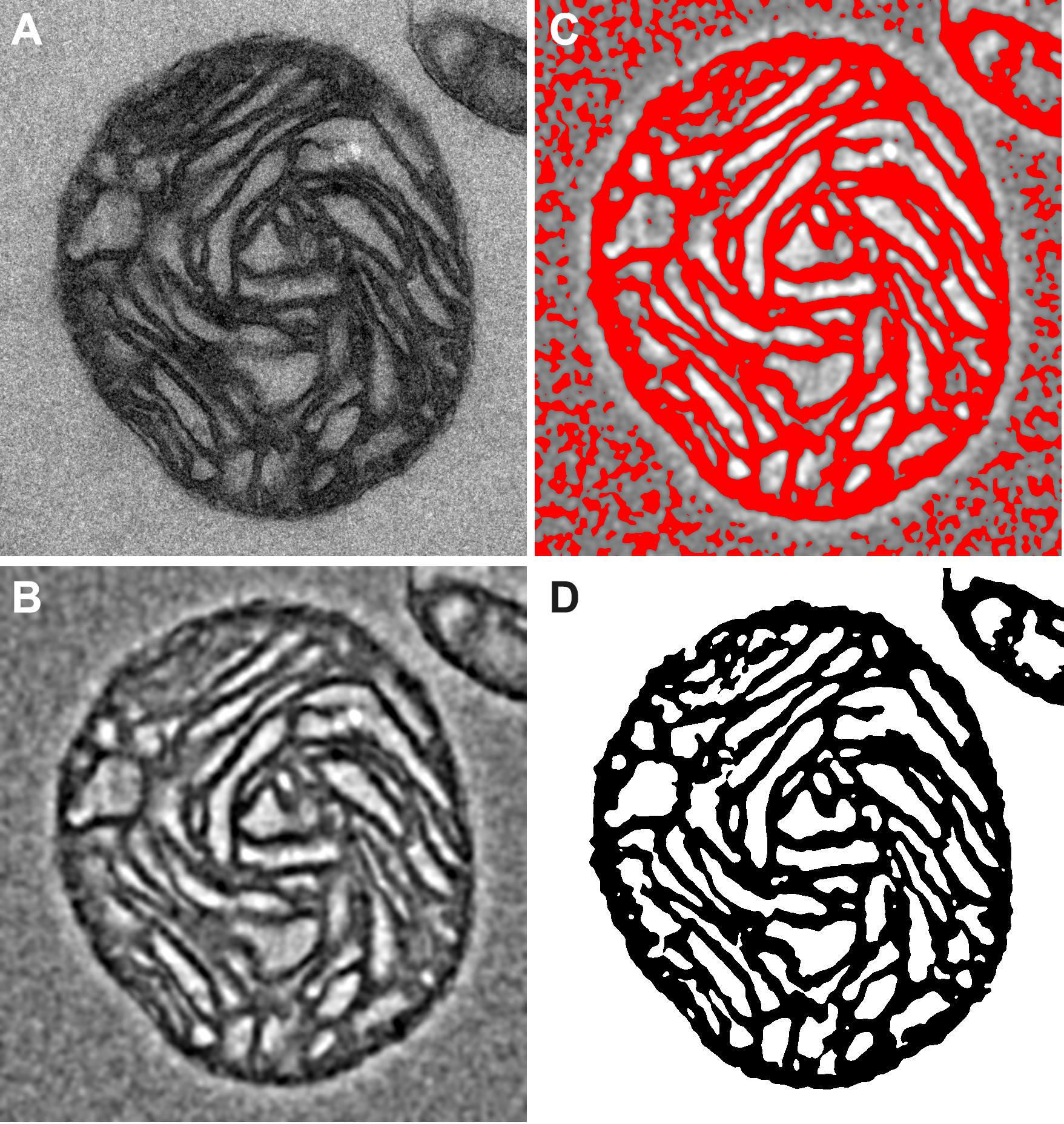

Supplement: S7 Fig — Example of the image-processing pipeline for mitochondrial membranes segmentation in EM micrographs. A—original; B—band pass filter; C—Otsu threshold; D—particle size filtering. (TIF) [file pone.0234653.s007.tif]
