# Supplementary material for: Hexokinase II dissociation alone cannot account for changes in heart mitochondrial function, morphology and sensitivity to permeability transition pore opening following ischemia
Source: PLoS One. 2020 Jun 24;15(6):e0234653. doi: 10.1371/journal.pone.0234653 (PMC7313731; doi:10.1371/journal.pone.0234653)
Supplement: S1 Table — Hearts were perfused according to the protocols described in the Material and Methods section. All the data presented in the table correspond to hemodynamic function recorded prior to the index ischemia at the end of the stabilization period. Data for each parameter were analyzed by a one-way ANOVA followed by Holm-Šídák pos-hoc test to correct for multiple comparisons. *, p<0.05 vs pre-ischemia or ischemia. Abbreviations: EDP—end-diastolic pressure; IPC—ischemic preconditioning; Isch.—ischemia; RRP; rate pressure product; SP—systolic pressure. (DOCX) [file pone.0234653.s008.docx]

Table S1 – Hemodynamic data monitored before ischemia of hearts used for mitochondrial isolation.

|  | Aortic Pressure (mmHg) | | Heart Rate (beats/min) | | EDP (mmHg) | | SP (mmHg) | | RPP (mmHg/min) | |
| --- | --- | --- | --- | --- | --- | --- | --- | --- | --- | --- |
|  | 20min | 35min | 20min | 35min | 20min | 35min | 20min | 35min | 20min | 35min |
| **Pre-Isch.** | 96.1±7.7 | 111.0±6.9 | 296±10 | 282±13 | 7.8±0.6 | 7.3±0.5 | 91.9±7.1 | 95.4±5.4 | 24931±2033 | 25907±1971 |
| **Isch.** | 104.3±8.6 | 115.4±5.3 | 260±13 | 268±20 | 6.8±0.6 | 5.4±0.8 | 111.8±6.9 | 116.4±5.7 | 27799±2853 | 30070±2710 |
| **IPC** | 97.3±5.5 | 52.9±2.0* | 278±15 | 282±12 | 7.8±0.6 | 7.5±0.6 | 110.5±5.4 | 80.5±2.8* | 28350±2012 | 20100±1309* |

Hearts were perfused according to the protocols described in the Material and Methods section. All the data presented in the table correspond to hemodynamic function recorded prior to the index ischemia at the end of the stabilization period. Data for each parameter were analyzed by a one-way ANOVA followed by Holm-Šídák pos-hoc test to correct for multiple comparisons. *, p<0.05 vs pre-ischemia or ischemia. Abbreviations: EDP – end-diastolic pressure; IPC – ischemic preconditioning; Isch. – ischemia; RRP; rate pressure product; SP - systolic pressure;
