# Supplementary material for: Hexokinase II dissociation alone cannot account for changes in heart mitochondrial function, morphology and sensitivity to permeability transition pore opening following ischemia
Source: PLoS One. 2020 Jun 24;15(6):e0234653. doi: 10.1371/journal.pone.0234653 (PMC7313731; doi:10.1371/journal.pone.0234653)
Supplement: S2 Table — All the data presented in the table were obtained during the index ischemia. T0 indicates time at which rigor started; Amax, rigor maximum amplitude. Data for each parameter was analyzed by a two-tail Student’s t test. *, p<0.05. Abbreviations: IPC—ischemic preconditioning; n.a.—not applicable. (DOCX) [file pone.0234653.s009.docx]

Table S2 – Parameters relating to ischemic contracture of hearts used for mitochondrial isolation.

|  | T_0_ (sec) | A_max_ (mmHg) |
| --- | --- | --- |
| **Pre-Ischemia** | n.a. | n.a. |
| **Ischemia** | 716.5 ± 42.8 | 58.1 ± 5.8 |
| **IPC** | 466.3 ± 14.9* | 80.3 ± 5.0* |

All the data presented in the table were obtained during the index ischemia. T_0_ indicates time at which rigor started; A_max_, rigor maximum amplitude. Data for each parameter was analyzed by a two-tail Student’s t test. *, p<0.05. Abbreviations: IPC – ischemic preconditioning; n.a. – not applicable.
