# Supplementary material for: Hexokinase II dissociation alone cannot account for changes in heart mitochondrial function, morphology and sensitivity to permeability transition pore opening following ischemia
Source: PLoS One. 2020 Jun 24;15(6):e0234653. doi: 10.1371/journal.pone.0234653 (PMC7313731; doi:10.1371/journal.pone.0234653)
Supplement: S3 Table — Hearts were perfused according to the protocols described in the Material and Methods section. All the data presented in the table correspond to hemodynamic function recorded after 5 and 60 min of reperfusion. Infarct size was assessed by the TTC staining 120min after starting reperfusion. Data for each parameter were analyzed by a two-tail Student’s t test. *, p < 0.05; ***, p < 0.001. Abbreviations: AAR—area at risk; IPC—ischemic preconditioning. (DOCX) [file pone.0234653.s010.docx]

Table S3 – Hemodynamic data monitored during reperfusion for the group of hearts used to study infarct size.

|  | Developed Pressure(% relative to 20min of stabilization) | | RPP(% relative to 20min of stabilization) | | Infarct Size (% AAR) |
| --- | --- | --- | --- | --- | --- |
|  | 5min | 60min | 5min | 60min |  |
| **Ischemia** | 5.80 ± 1.25 | 26.0 ± 1.4 | 14.8 ± 4.0 | 26.4 ± 2.6 | 24.7 ± 2.0 |
| **IPC** | 26.8 ± 11.0 | 61.5 ± 11.6* | 26.2 ± 8.1 | 56.6 ± 11.4* | 5.2 ± 0.7*** |

Hearts were perfused according to the protocols described in the Material and Methods section. All the data presented in the table correspond to hemodynamic function recorded after 5 and 60 min of reperfusion. Infarct size was assessed by the TTC staining 120min after starting reperfusion. Data for each parameter were analyzed by a two-tail Student’s t test. *, p < 0.05; ***, p < 0.001. Abbreviations: AAR – area at risk; IPC – ischemic preconditioning.
