# Supplementary material for: Hexokinase II dissociation alone cannot account for changes in heart mitochondrial function, morphology and sensitivity to permeability transition pore opening following ischemia
Source: PLoS One. 2020 Jun 24;15(6):e0234653. doi: 10.1371/journal.pone.0234653 (PMC7313731; doi:10.1371/journal.pone.0234653)
Supplement: S4 Table — Data are presented as mean ± SD and differences relatively to baseline were analyzed by a one-way ANOVA followed by Dunnet’s pos-hoc test to correct for multiple comparisons. ***, p<0.001 vs baseline. (DOCX) [file pone.0234653.s011.docx]

Table S4 – Morphometric data from EM micrographs of isolated mitochondria incubated with different ANT ligands.

|  |  |  |  |  |  |  |
| --- | --- | --- | --- | --- | --- | --- |
|  | **Number**  **of mitoch.** | **Total Area**  (μm^2^) | **Perimeter**  (μm) | **Circularity** | **Aspect Ratio** | **Total membranes perimeter**  (μm) |
| **Baseline** | 193 | 0.722 ± 0.406 | 3.12 ± 0.81 | 0.870 ± 0.079 | 1.30 ± 0.33 | 6.33 ± 4.32 |
| **Ca^2+^** | 347 | 0.710 ± 0.403 | 3.11 ± 0.87 | 0.859 ± 0.103 | 1.35 ± 0.36 | 8.76 ± 6.49 *** |
| **ADP** | 290 | 0.886 ± 0.428 *** | 3.47 ± 0.79 *** | 0.876 ± 0.074 | 1.29 ± 0.25 | 7.29 ± 5.26 |
| **CAT** | 335 | 0.744 ± 0.379 | 3.16 ± 0.78 | 0.881 ± 0.070 | 1.24 ± 0.26 | 8.98 ± 7.30 *** |

Data are presented as mean ± SD and differences relatively to baseline were analyzed by a one-way ANOVA followed by Dunnet’s pos-hoc test to correct for multiple comparisons. ***, p<0.001 vs baseline.
